# Risk Prediction and Management for Central Nervous System Infection After Resection for Gliomas—The 8-Year Experience from a Tertiary Medical Center

**DOI:** 10.3390/jcm13247733

**Published:** 2024-12-18

**Authors:** Xin Zhang, Zhiyao Zheng, Xiaopeng Guo, Hai Wang, Le Gong, Yu Wang, Fuping Guo, Wenbin Ma

**Affiliations:** 1 Department of Neurosurgery, Peking Union Medical College Hospital, Chinese Academy of Medical Sciences, Peking Union Medical College, Beijing 100006, China; sanjinbs@163.com (X.Z.);; 2 Department of Infectious Diseases, Peking Union Medical College Hospital, Chinese Academy of Medical Sciences, Peking Union Medical College, Beijing 100006, China

**Keywords:** glioma, central nervous system infection, risk factors, antibiotic

## Abstract

**Objective**: To identify risk factors for central nervous system infection (CNSI) following glioma resection and develop a predictive model. **Methods**: Retrospective analysis of 435 glioma resection cases was conducted to assess CNSI risk factors. A nomogram predictive model was constructed and validated internally and externally. CSF characteristics and antibiotic use in CNSI patients were summarized and the impact of CNSI on long-term prognosis was evaluated. **Results**: CNSI incidence was 14.9%. Independent risk factors included ventricular opening, postoperative systemic infection, maximum diameter ≥ 5 cm, and preoperative peripheral blood monocyte percentage ≥ 10%. The predictive model showed good performance (C statistic = 0.797, AUC = 0.731). CNSI patients had elevated CSF protein and leukocytes, with meropenem and vancomycin as primary antibiotics. CNSI had no significant impact on long-term prognosis. **Conclusions**: Key risk factors for CNSI were identified, and an effective predictive model was established, providing important references for clinical decision-making and CNSI management.

## 1. Introduction

Central nervous system infection (CNSI) is a potential complication following neurosurgical procedures, with an incidence ranging from 0.8% to 25.0% [1,2,3,4]. As the most common primary malignant brain tumor [5], glioma treatment primarily involves maximal safe resection, followed by adjuvant radiotherapy and chemotherapy, as indicated [6]. Unlike meningioma surgery, glioma surgery involves manipulation of brain parenchyma, resulting in longer operation times and potentially increased risk of CNSI due to postoperative corticosteroid use [7,8]. CNSI not only prolongs hospital stays and increases healthcare costs but also may delay the initiation of radiotherapy and chemotherapy, raising mortality risk and the incidence of neurological deficits [9,10]. Therefore, this study aims to investigate high-risk factors for CNSI in glioma patients postoperatively, including clinical characteristics, laboratory test results, and surgery-related data, and to construct a risk prediction model. Additionally, this study will summarize the cerebrospinal fluid (CSF) characteristics and antibiotic usage in glioma patients with postoperative CNSI and explore the impact of CNSI on prognosis.

## 2. Methods

### 2.1. Patient Identification

This study was conducted at Peking Union Medical College Hospital. A total of 435 glioma patients who underwent tumor resection from January 2015 to March 2023 were consecutively enrolled. After excluding patients who underwent biopsy and those with missing data, 337 patients were included in the final analysis, including 114 glioblastomas, 89 astrocytomas, 73 oligodendrogliomas, 22 gangliogliomas, and 39 other types of gliomas. This is a retrospective clinical study. Although informed consent was not obtained from all patients, the study was approved by the Ethics Review Committee of Peking Union Medical College Hospital (Ethical Code: K23C1427).

### 2.2. Data Extraction

The diagnosis of CNSI was based on the diagnostic criteria defined by the Centers for Disease Control and Prevention (CDC) [10]. Specifically, these criteria include:

(1) A definitive diagnosis of CNSI can be established when the CSF culture is positive for microorganisms.

(2) Even if the CSF culture is negative, the diagnosis of CNSI can still be confirmed if the patient exhibits the following clinical signs or symptoms: (a) fever (>38 °C); (b) headache; (c) meningeal irritation signs; (d) increased CSF white blood cell (WBC) count; (e) elevated CSF protein levels and/or decreased glucose levels; (f) detection of pathogens in CSF samples by Gram staining; and (g) positive blood cultures for microorganisms. A definitive diagnosis is established by meeting at least one condition from (a, b, c) and at least one additional condition from (d, e, f, g).

A standardized database was established, from which clinical information was extracted from the patients’ medical records. The preoperative patient information recorded included age, gender, BMI, and the following clinical features: whether it was a primary glioma, the presence of diabetes or other systemic diseases, history of radiotherapy and chemotherapy, corticosteroid use, the presence of other systemic infections, whether the tumor exhibited necrosis, the maximum tumor diameter, and the levels of immune-related cells in the routine blood test upon admission. Intraoperative clinical features included the duration of surgery, the opening of the ventricle and frontal sinus, and the placement of external drainage. Postoperative clinical features involved the duration of external drainage placement, the occurrence of other systemic infections, seizures, corticosteroid use, the maximum diameter of the tumor cavity, and whether multiple surgeries were performed (Table 1).

### 2.3. Statistical Analysis

Data analysis was performed using R language (version 4.2.3), and the relevant code can be found in the Supplementary File. Comparison of continuous variables was performed using the Student’s *t*-test or the Mann–Whitney U test, and comparison of categorical variables was performed using the Fisher exact test (Table 1). Variables with a *p*-value < 0.05 in Table 1 were included in multivariate logistic regression analysis to calculate the odds ratio (OR) and 95% confidence interval (CI) for the identification of independent risk factors for CNSI. Additionally, variables exhibiting a *p*-value < 0.05 in the multivariate logistic regression analysis were set for constructing the predictive model (Table 2). The cohort was randomly divided, with 85% of patients (n = 286) assigned to the training set and the remaining 15% (n = 51) to the external validation set. The predictive model was accompanied by a nomogram to visually estimate the probability of postoperative CNSI. Model performance was evaluated in terms of discrimination (C-statistic) and calibration (calibration curve). To reduce overfitting and quantify optimism bias, internal validation was performed using 1000 bootstrap resampling, and the optimized corrected C-statistic was calculated. External validation was performed using ROC curves. Decision curve analysis (DCA) was used to assess the clinical effectiveness and net benefit of the nomogram. Data for CSF protein, glucose, white blood cells, etc., in CNSI patients were expressed as median and interquartile range (IQR). The duration of antibiotic treatment was presented as the mean. Kaplan–Meier survival analysis was used to explore the impact of CNSI on the prognosis of glioma patients undergoing surgery.

## 3. Results

### 3.1. Study Population and Surgical Characteristics

This study included 337 patients who met the inclusion criteria. Table 1 presents their baseline characteristics and statistically significant variables. Among the patients, 43.3% were female, and 61 of them were diagnosed with CNSI postoperatively. The median age was 47 years (IQR: 34–57) in the group without CNSI and 46 years (IQR: 34–55) in the CNSI group. Both groups had a high proportion of primary glioma patients (83.70% and 83.61%, respectively). In terms of surgery, the median duration of surgery was 5 h (IQR: 4.0–6.3) in the group without CNSI, with a ventricle opening rate of 17.39%, an external drainage placement rate of 34.42%, and a frontal or ethmoid sinus opening rate of 6.52%. In the CNSI group, the median duration of surgery was 5.5 h (IQR: 4.7–7.0), with a ventricle opening rate of 47.54%, an external drainage placement rate of 60.66%, and a frontal or ethmoid sinus opening rate of 6.56%.

### 3.2. Risk Factors for Postoperative CNSI

Table 1 summarizes the variables assessed for the risk of postoperative CNSI. Statistically significant correlations (*p*-value < 0.05) were found for the preoperative percentage of peripheral blood monocytes, maximum tumor diameter, duration of surgery, ventricle opening, insertion of catheter into the tumor cavity, duration of external drainage, postoperative other systemic infections, maximum cavity diameter, and multiple hospitalizations. Multivariate logistic regression analysis identified independent risk factors as ventricle opening (OR 2.97, *p* < 0.01), postoperative other systemic infections (OR 4.03, *p* = 0.01), increased maximum cavity diameter (OR 1.03, *p* = 0.02), and elevated preoperative percentage of monocytes (OR 1.19, *p* = 0.04) (Table 2).

### 3.3. Model Development and Performance Validation

**Figure 1:**
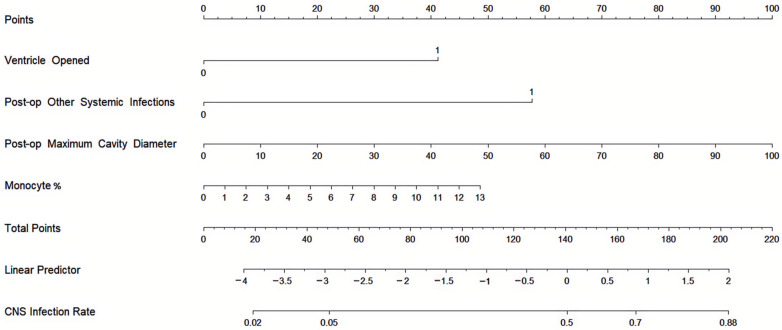
Nomogram for predicting the probability of postoperative CNSI in glioma patients. This nomogram is constructed based on independent risk factors for postoperative CNSI, providing a visual estimate of the CNSI probability. To use the nomogram, add up the corresponding scores of each variable on the scales to obtain the total score, then draw a vertical line from the total score scale to the probability axis to read the corresponding probability of CNSI.

**Figure 2:**
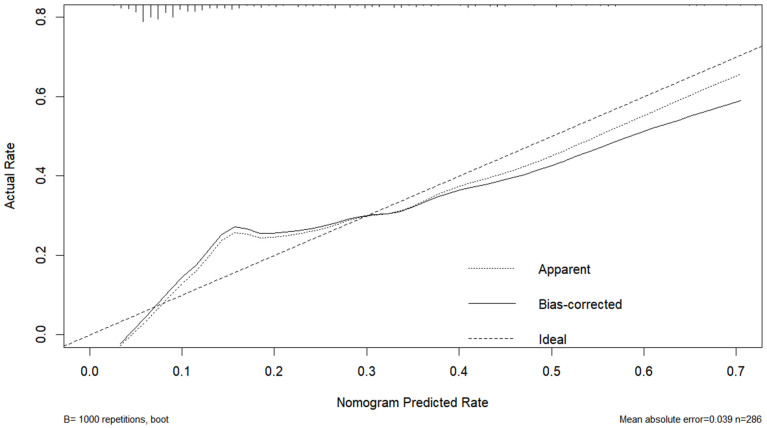
Internal calibration curve. In an ideal scenario, a perfectly accurate predictive model would have observed probabilities that align with predicted probabilities, distributed along the 45° line (Ideal). The Apparent line in the figure represents the apparent calibration curve of the model on the development dataset, while the solid line demonstrates the calibration result after bias correction through 1000 bootstrap resampling, which is closer to the ideal state.

**Figure 3:**
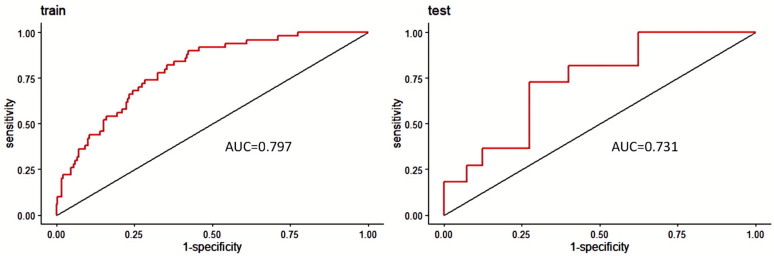
ROC curve. The red curve and the area under the curve (AUC) respectively demonstrate the overall performance of the predictive model on the training set and the test set. The model exhibited good performance in internal validation (AUC = 0.797) and also demonstrated robust predictive ability in the external validation set (AUC = 0.731).

**Figure 4:**
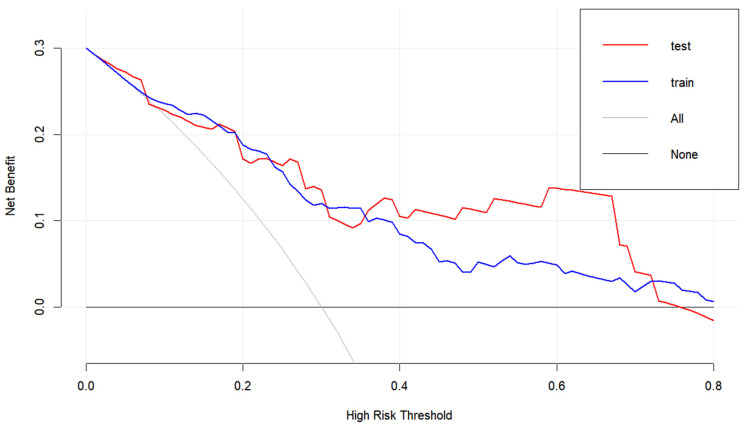
Decision curve analysis (DCA) illustrating the clinical value of the predictive model. The *y*-axis represents the net benefit, and the bold black line represents the strategy of predicting CNSI risk based on the nomogram. The gray solid line represents the scenario where all patients are assumed to develop CNSI, while the black, thin, solid line represents the scenario where no patients are assumed to develop CNSI. The DCA shows that using the nomogram prediction strategy provides greater net benefit within a threshold risk range of 0 to 30%, confirming the clinical utility of the model.

Based on the first four variables from Table 2, we developed a nomogram to predict the probability of postoperative CNSI in patients. The C-statistic of the prediction model was 0.797, indicating good discrimination (Figure 1). The calibration curve further confirmed the model’s excellent performance in terms of prediction accuracy and discrimination, with specific indicators of mean absolute error 0.038, mean square error 0.00228, and absolute error quantile 0.085 (Figure 2). External validation using the ROC curve yielded an AUC value of 0.731 for the model (Figure 3). DCA showed positive net benefit with a threshold risk range of 0 to 30% (Figure 4).

### 3.4. CSF Characteristics of Patients with CNSI

The CSF characteristics of patients with central nervous system infection were as follows: median protein content was 1.73 g/L (IQR: 1.03–3.06), median glucose content was 2.7 mmol/L (IQR: 1.90–3.70), median chloride content was 120 mmol/L (IQR: 116.00–122.00), median white blood cell count was 1478 × 10^6^/L (IQR: 467 × 10^6^–4204.25 × 10^6^), and median percentage of polymorphonuclear cells was 84.1% (IQR: 74.55–90.83%) (Table 3).

### 3.5. Microbiological Characteristics of Central Nervous System Infection and Antibiotic Use

Among the 61 patients with central nervous system infection, only three had positive microbial culture results. The identified pathogens included Staphylococcus hemolyticus, Staphylococcus epidermidis, and Acinetobacter baumannii (Table 4).

A total of 50 patients received antibiotic treatment. Vancomycin was most commonly used against Gram-positive bacteria, with 43 administrations and an average treatment course of 7.79 days. Meropenem was most commonly used against Gram-negative bacteria, with 27 administrations and an average treatment course of 8.41 days (Table 5).

### 3.6. Long-Term Follow-Up and Survival Analysis

**Figure 5:**
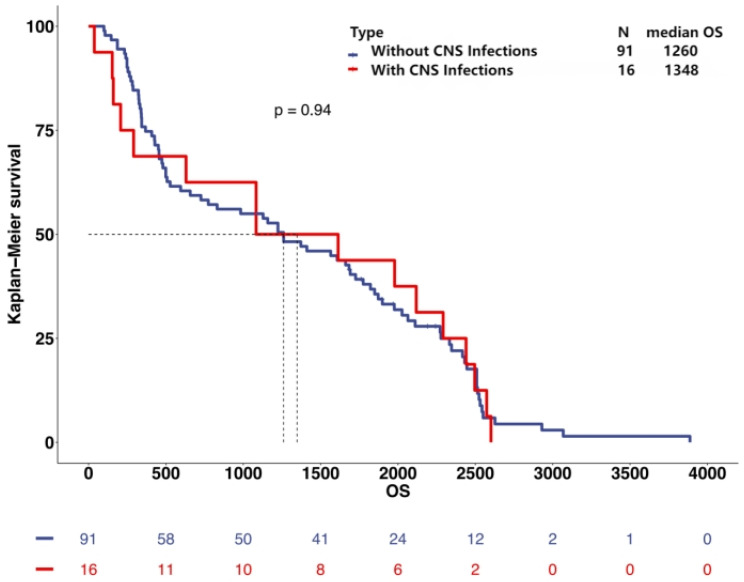
Kaplan–Meier survival analysis of patients with and without CNSI (Unit: days).

This study involved long-term follow-up of the included patients, obtaining survival data for 107 patients. Among 107 patients, 91 patients did not develop CNSI (median survival time of 1260 days) and 16 patients developed CNSI (median survival time of 1348 days). Kaplan–Meier survival analysis showed no significant difference in prognosis between the two groups (Figure 5).

## 4. Discussion

### 4.1. Risk Factors and Predictive Model

Multivariate logistic regression analysis revealed that ependymal opening, postoperative systemic infection, postoperative maximum cavity diameter, and percentage of monocytes were independent risk factors for CNSI following glioma resection. Ependymal opening is commonly observed when the tumor invades the ependyma or adjacent brain tissue, a characteristic unique to intraparenchymal tumors. Since the goal of glioma surgery is to maximize tumor resection, it may inadvertently damage the ependyma. Currently, there is no literature reporting the risk of ependymal opening in glioma surgery and postoperative CNSI. However, the results suggest that the opening of the ependyma is an independent risk factor for postoperative CNSI. Therefore, when ependymal opening occurs during glioma surgery and patients present with symptoms such as fever, headache, and meningeal signs, timely lumbar puncture and antibiotic treatment should be considered. Additionally, minimizing ependymal wall damage, whenever possible, may be a more reasonable approach.

Postoperative extracranial CNSI mainly includes respiratory and urinary tract infections. Among the 337 patients, 14 developed pneumonia and 5 had urinary tract infections. Notably, eight patients with pneumonia and one with urinary tract infection subsequently presented with meningitis-related symptoms and were diagnosed with CNSI. The most common pathogen in pneumonia complicated with CNSI is Streptococcus pneumoniae [1,11,12,13,14,15], which often colonizes the nasopharynx and can cause pneumonia in the presence of immunodeficiency. It is highly invasive and can disrupt the blood–brain barrier (BBB) and enter the CSF upon entering the bloodstream, leading to CNSI [11,12]. R A Hirst’s study [12] confirmed that the same Streptococcus pneumoniae strain could be cultured from patients with pneumonia and CNSI. Studies on urinary tract infection complicated with CNSI are mostly conducted in infants, with a very low incidence (approximately less than 0.1%) [16,17,18,19,20], which may be related to the specific pathogenic mechanism of uropathogenic Escherichia coli (UPEC), the flushing action of urine, and the strong immune function of the uroepithelium [16,21,22]. Therefore, patients with postoperative pneumonia need to strengthen respiratory management and monitor for symptoms of CNSI.

As an indicator of the residual cavity volume following tumor resection [23,24], the maximum postoperative cavity diameter has not been explicitly reported in the literature to be directly associated with the development of CNSI in glioma patients. However, from a pathophysiological perspective, it is speculated that a larger postoperative residual cavity volume may indirectly reflect the complexity and invasiveness of the surgery, such as longer operative time, more extensive exposure of brain tissue, greater surgical trauma, and severe disruption of the CSF circulation, all of which may potentially increase the risk of CNSI. Notably, the maximum preoperative tumor diameter has not been confirmed as an independent risk factor for CNSI, which may be related to the principle of functional preservation followed in glioma surgery [6,25] and the phenomenon of brain displacement [26], as the residual cavity volume is not always directly correlated with tumor size. From our surgical experience, the probability of CNSI following surgery varies among different types of brain tumors. For example, meningiomas are much less likely to develop CNSI postoperatively compared to gliomas, which may be attributed to the fact that meningioma surgery usually does not involve manipulation of brain parenchyma and has a relatively shorter operative time due to its superficial location.

This study confirms that the percentage of peripheral blood monocytes before surgery is an independent risk factor for CNSI following glioma resection. Monocytes play a key role in the immune response, participating in the production of pro-inflammatory mediators such as interleukins, tumor necrosis factor, and interferon [27,28]. Peripheral monocytes can migrate to the brain under the chemotactic influence of inflammatory mediators in the cerebrospinal fluid, where they either directly participate in the inflammatory response or differentiate into microglia, thereby exacerbating inflammation [29,30]. In the context of Salmonella-associated CNSI, studies have found that monocytes have higher levels of accumulation and infiltration in brain tissue, while the levels of neutrophils, dendritic cells, T cells, B cells, and natural killer cells are relatively low [31], further suggesting the important role of monocytes in the pathophysiology of CNSI.

Although the duration of surgery, placement of external drainage tubes, and the maximum preoperative tumor diameter were not confirmed as independent risk factors for postoperative CNSI in the multivariate analysis, the results of univariate analysis still suggest their association with the risk of CNSI. Therefore, in clinical practice, these factors still need to be appropriately considered.

In contrast to previous studies [7,32,33], this study did not find a significant association between diabetes and the use of corticosteroids with postoperative CNSI. This discrepancy may be attributed to the high usage rate of corticosteroids and the low proportion of diabetic patients in this study. At our center, dexamethasone is routinely administered within 7 days after surgery to manage peritumoral brain edema and related neurological deficits, maintain patients’ good mental status, and promote early recovery and postoperative rehabilitation [34]. Only 18 patients in the study cohort had diabetes.

In terms of model development, this study is the first to predict the risk of postoperative CNSI in glioma patients, incorporating variables that are easily accessible and quantifiable for neurosurgical clinicians. The developed nomogram provides an intuitive scoring system to estimate the probability of CNSI, demonstrating good discriminative ability after internal and external validation. Additionally, DCA indicates that within the risk threshold range of 0–30%, the intervention decision based on this predictive model yields significant benefits. Potential interventions include, but are not limited to, extending the duration of postoperative prophylactic antibiotic use and upgrading the level of prophylactic antibiotics during and after surgery.

### 4.2. CSF Characteristics and Antibiotic Use

Elevated protein levels, decreased glucose levels, and increased white blood cell count in CSF often indicate bacterial CNSI [35]. The median CSF glucose level in patients in this cohort was close to normal, possibly due to higher blood glucose levels in some patients during lumbar puncture. Among patients with positive CSF microbiological cultures, one case each of Staphylococcus haemolyticus, Staphylococcus epidermidis, and Acinetobacter baumannii was identified. Of these three patients, two developed severe CNSI (subdural empyema) and underwent thorough debridement and drainage, including removal of the skull bone flap. Another patient received a 14-day course of antibiotic therapy, exceeding the duration for patients with negative CSF cultures. Staphylococcus haemolyticus and Staphylococcus epidermidis are common pathogens of nosocomial infections [36,37,38] and are generally susceptible to most antibiotics for Gram-positive bacteria whereas Acinetobacter baumannii is multidrug-resistant, posing greater challenges for treatment.

The treatment strategy should be based on the specific antibiotic susceptibility profile of the isolated strain. Among the 61 patients diagnosed with CNSI, 50 received antibiotic treatment, 5 did not receive antibiotics due to the decision of the attending physician, and 6 were excluded from the analysis due to complex antibiotic use. Vancomycin is the first-choice antibiotic for Gram-positive bacterial infections, while meropenem and cefoperazone are commonly used for Gram-negative bacterial infections, with the combination of meropenem and vancomycin being the most frequent. Meropenem is suitable for severe patients or infections that do not respond to other antibiotics, and it demonstrates superior efficacy compared to ceftriaxone and ceftazidime [39,40]. Our criteria for discontinuing antibiotics include normal body temperature, resolution of meningeal irritation signs, CSF white blood cell count < 100/L, and normalization of protein and glucose levels.

Some patients developed symptoms suggestive of CNSI after surgery, with bloody CSF on lumbar puncture and CSF cytological analysis showing significant increases in both white blood cells and red blood cells. This may lead to difficulties for inexperienced clinicians in differentiating whether the symptoms are caused by the stimulation of the nervous system by bloody CSF or by concurrent CNSI during the differential diagnosis. Compared to subarachnoid hemorrhage caused by aneurysm rupture, the latter is characterized by a significant increase in red blood cell count with rare abnormal elevation of white blood cell count in the CSF [41,42]. For example, the CSF sample of a patient with subarachnoid hemorrhage had a red blood cell count of 45 × 10^6^/L, while the white blood cell count was only 0.104 × 10^6^/L. Therefore, when both red blood cell and white blood cell counts are elevated in the CSF, the possibility of CNSI should be considered first.

### 4.3. Survival Analysis

Given that patients with glioma require radiotherapy and chemotherapy after surgery, postoperative CNSI may delay the initiation of these treatments. Therefore, this study explored whether CNSI affects the prognosis of patients with glioma. Among the 337 patients, 107 provided survival data, of which 91 did not develop CNSI and 16 did. Kaplan–Meier survival analysis showed no significant difference in survival rates between the two groups, demonstrating that postoperative CNSI does not affect the overall survival of patients with glioma. Leif-Erik Bohman’s study, which included 382 patients with glioblastoma, found no significant difference in overall survival between the infection group and the control group [43]. However, Pasquale De Bonis’ study showed that among 197 patients with glioblastoma, the median survival of the postoperative infection group was significantly prolonged (30 months vs. 15 months) [44], with possible mechanisms, including immune system activation, induced by infection and the competitive effect of tumor cells and bacteria for survival space and resources in the local environment. The specific mechanisms require further research, which may provide new ideas for experimental studies using genetically modified bacteria to treat glioma.

## 5. Limitation

This study is a single-center retrospective analysis and only 107 patients had available survival data for survival analysis, which may limit the generalizability and statistical power of the results. Additionally, although the predictive model developed showed good performance in both the training set and the test set, its clinical application still requires further validation. To this end, we plan to conduct a prospective cohort study to optimize the predictive model and explore potential strategies for reducing the incidence of postoperative CNSI in patients with glioma in clinical practice. The study will focus on high-risk patients for CNSI, evaluating the effectiveness of extending the duration of prophylactic antibiotic use or upgrading the level of antibiotics in reducing the incidence of CNSI. Further analyses will include stratified studies based on tumor histological type and grade to clarify the impact of postoperative CNSI on patient prognosis.

## 6. Conclusions

This study identified multiple parameters predictive of CNSI after glioma resection and developed a superior predictive model. We summarized the CSF characteristics of patients with CNSI, providing important references for the diagnosis of CNSI in cases with negative CSF microbiological cultures. Furthermore, the study demonstrated that meropenem is an effective drug for treating CNSI after glioma resection. Additionally, we found that CNSI did not affect the prognosis of patients with glioma. These findings will provide strong support for neurosurgeons in personalized prevention, diagnosis, and treatment of CNSI after glioma resection.

## Figures and Tables

**Table 1:** Comparison of the 2 groups regarding the development of CNSI. Continuous variable data are presented with n (%), while categorical variable data are represented by the median [IQR]. Abbreviation: BMI = body mass index; Pre-op = pre-operation; Post-op = post-operation; * = *p* value < 0.05.

Variable	No CNSI	CNSI	*p* Value
No. of Procedures	276	61	
Age (yrs)	47.00 [33.75, 57.00]	46.00 [34.00, 55.00]	0.67
Female Sex	123 (44.57%)	23 (37.70%)	0.40
BMI	24.03 [20.29, 27.77]	24.76 [21.03, 28.49]	0.17
Primary Glioma	231 (83.70%)	51 (83.61%)	1.00
Diabetes mellitus	16 (5.80%)	2 (3.28%)	0.63
Comorbidities in Other Systems	113 (40.94%)	27 (44.26%)	0.74
Pre-op Radiotherapy	40 (14.49%)	12 (19.67%)	0.41
Pre-op Chemotherapy	36 (13.04%)	12 (19.67%)	0.26
Pre-op Steroid Use	76 (27.54%)	16 (26.23%)	0.96
Pre-op Concomitant Organ Infections	3 (1.09%)	3 (4.92%)	0.13
Pre-op Tumor Necrosis	173 (62.68%)	41 (67.21%)	0.60
Maximum Tumor Diameter (mm)	41.60 [32.15, 55.00]	49.50 [40.00, 60.00]	<0.01 *
Surgery Duration (hours)	5.00 [4.00, 6.30]	5.50 [4.70, 7.00]	0.01 *
Ventricle Opened	48 (17.39%)	29 (47.54%)	<0.01 *
Frontal/Ethmoid Opened	18 (6.52%)	4 (6.56%)	1.00
Tumor Cavity Catheter Insertion	95 (34.42%)	37 (60.66%)	<0.01 *
External Drain Duration (days)	0.00 [0.00, 1.00]	1.00 [0.00, 3.00]	<0.01 *
Post-op Other Systemic Infections	10 (3.62%)	11 (18.03%)	<0.01 *
Post-op Seizures	16 (5.80%)	3 (4.92%)	1.00
Post-op Steroid Use	254 (92.03%)	59 (96.72%)	0.31
Post-op Maximum Cavity Diameter (mm)	43.15 [33.77, 54.82]	53.90 [46.90, 63.70]	<0.01 *
Multiple Hospital Surgeries	1 (0.36%)	4 (6.56%)	<0.01 *
Pre-op Blood Cell Tests			
Absolute WBC Count (×10^9^/L)	6.20 [5.19, 7.74]	6.53 [4.94, 8.17]	0.57
Absolute Lymphocyte Count (×10^9^/L)	1.74 [1.34, 2.22]	1.68 [1.32, 2.12]	0.38
Lymphocyte %	28.79 [18.92,38.66]	27.20 [18.43,35.97]	0.25
Absolute Monocyte Count (×10^9^/L)	0.35 [0.28, 0.43]	0.37 [0.30, 0.47]	0.14
Monocyte %	5.60 [4.68, 6.50]	6.30 [4.90, 6.90]	0.03 *
Absolute Neutrophil Count (×10^9^/L)	3.75 [2.83, 4.87]	3.70 [2.89, 5.42]	0.48
Neutrophil %	60.80 [54.05, 68.82]	62.60 [56.00, 69.60]	0.24
Absolute Eosinophil Count (×10^9^/L)	0.09 [0.05, 0.15]	0.08 [0.05, 0.14]	0.76
Eosinophil %	1.50 [0.80, 2.50]	1.50 [0.80, 2.60]	0.94
Absolute Basophil Count (×10^9^/L)	0.03 [0.02, 0.03]	0.02 [0.02, 0.04]	0.97
Basophil %	0.40 [0.30, 0.60]	0.40 [0.20, 0.60]	0.83

**Table 2:** Multivariate analysis for the risk for CNSI; * = *p* value < 0.05.

Variable	OR	CI	*p* Value
Ventricle Opened	2.97	1.54–5.71	<0.01 *
Post-op Other Systemic Infections	4.03	1.34–12.14	0.01 *
Post-op Maximum Cavity Diameter	1.03	1.01–1.06	0.02 *
Pre-op Blood Monocyte %	1.19	1.01–1.41	0.04 *
Tumor Cavity Catheter Insertion	1.63	0.66–4.02	0.29
External Drain Duration	1.07	0.84–1.38	0.57
Maximum Tumor Diameter	1.00	0.97–1.02	0.72
Surgery Duration	0.96	0.81–1.15	0.66
Multiple Hospital Surgeries	8.10	0.73–89.54	0.09

**Table 3:** CSF characteristics of the 61 patients who had CNSI.

Variable	Median	IQR	Reference
Protein (g/L)	1.73	[1.03, 3.06]	0.15–0.45
Glucose (mmol/L)	2.7	[1.90, 3.70]	2.4–4.5
Chloride (mmol/L)	120	[116.00, 122.00]	120–132
WBC (10^6^/L)	1478	[467, 4204.25]	0–8
Multinucleated Cell %	84.1	[74.55, 90.83]	<70

**Table 4:** Pathogenic culture and drug sensitivity results of cerebrospinal fluid.

Bacteria	Sensitive Antibiotics	Resistant Antibiotics
Staphylococcus haemolyticus	Gentamicin, Linezolid, Selectrin, Teicoplanin, Vancomycin	Ciprofloxacin, Oxacillin, Erythromycin, Penicillin G
Staphylococcus epidermidis	Gentamicin, Linezolid, Vancomycin, Rifampicin, Selectrin, Teicoplanin	Oxacillin, Penicillin G
Acinetobacter baumanii	Minocycline, Tigecycline	Amikacin, Ceftazidime, Ciprofloxacin, Levofloxacin, Cefperazone-Sulbactam, Meropenem, Selectrin, Sulbactam-Ampicillin, Doxycycline, Cefepime, Imipenem, Tobramycin, Piperacillin-Tazobactam

**Table 5:** Antibiotic use in patients with CNSI.

	Antibiotic Varieties	Frequency of Use (n = 50)	Average Time of Use (Days)
Gram-positive	Vancomycin	43	7.79
	Linezolid	4	7.75
Gram-negative	Meropenem	27	8.41
	Cefperazone	17	6.88
	Ceftriaxone	6	6.83
	Ceftazidime	7	8.43
Common Antibiotic Combinations	Meropenem + Vancomycin	23	8.13
	Cefperazone + Vancomycin	8	6.13

## Data Availability

The datasets analyzed during the current study can be found in Supplementary Materials.

## Supplementary Materials

The following supporting information can be downloaded at: https://www.mdpi.com/article/10.3390/jcm13247733/s1, Table S1: dataset; Table S2: train; Table S3: test; R code S1: pred modle.
